# Comparison of the efficacy of Diclofenac 0.1% and Nepafenac 0.1% on anterior chamber cells in patients undergoing cataract surgery: A prospective clinical practice trial

**DOI:** 10.12669/pjms.39.5.6862

**Published:** 2023

**Authors:** Adnan Ahmad, Shams Ul Haq, Jamal Hussain, Javed Rasul

**Affiliations:** 1 Dr. Adnan Ahmad (FCPS, FRCS [UK], FICO (UK). Associate Professor, Dept. of Eye, Nowshera Medical College, Qazi Hussain Medical Complex, Nowshera, Pakistan; 2 Dr. Shams Ul Haq (FCPS). Consultant Ophthalmologist,District Headquarter Hospital, Timergara, Lower Dir, Pakistan; 3 Dr. Jamal Hussain (FCPS). Consultant Ophthalmologist,District Headquarter Hospital, Timergara, Lower Dir, Pakistan; 4 Dr. Javed Rasul (FCPS). Assistant Professor, Dept. of Eye, Pak International Medical College, Peshawar, Pakistan

**Keywords:** Anterior chamber inflammation, Aqueous cells, Cataract surgery, Topical nepafenac 0.1%, Topical diclofenac 0.1%

## Abstract

**Objectives:**

To compare the efficacy of topical Nepafenac 0.1 % and Diclofenac 0.1% eye drops in reducing the aqueous cells in the anterior chamber in an un-eventful post cataract surgery.

**Methods:**

This prospective, clinical trial was conducted at an Eye OPD of Qazi Hussain Ahmad Medical Complex, Nowshera from January till December 2021. Ophthalmic assessment included Visual acuity (VA), slit-lamp examination, Intraocular pressure (IOP), Central macular thickness (CMT) by Optical coherence tomography (OCT) and anterior chamber-aqueous cells measurement pre-operatively and at day 1^st^, 2^nd^, 4^th^ and 8^th^ week post-operatively. Patients were randomly allocated to topical diclofenac 0.1% (TD) four times a day and nepafenac 0.1% (TN) three times a day for four weeks each along with topical steroids and antibiotics.

**Results:**

Seventy patients (70) were randomly distributed into two treatment arms of 35 each. In both the arms VA improved which achieved a level of statistical significance post-operatively, however statistically insignificant difference was observed between the groups at 8^th^ week follow up visit (p= 0.62). However, IOP and CMT values didn’t achieve statistical significance between the arms pre and post operatively. In TN arm, level of AC-cells at 2^nd^ and 4^th^ week post-operatively were significantly lower (10.54 ± 4.05 and 08.20 ± 4.44) than TD arm (11.28 ± 5.04 and 09. 66 ± 5.50) with statistically significant difference (p < 0.05).

**Conclusions:**

Topical Nepafenac 0.1% was more effective in suppressing the anterior chamber inflammation as compared to diclofenac during the early few post-operative weeks.

## INTRODUCTION

Phacoemulsification is commonly employed procedure for cataract removal globally. The latest advancement in the phacoemulsification mechanics have considerably mitigated the adverse visual outcomes and enhanced its safety but still complications do occur. Cystoid macular edema (CME), with an estimated occurrence between 0.2 % and 4.1 %, causes of sub-optimal visual acuity (VA) after an un-eventful cataract extraction^1^,^2^ post-operative inflammatory reaction is one of the most important causes for CME, mainly due to the disruption of the inner blood retinal barrier by the inflammatory mediators dispersed inside the eye.^3^ There is release of inflammatory markers during cataract surgery in the anterior chamber which permeates into the vitreous and finally reaches the retinal vascular bed. There is increased vascular permeability especially in the peri-foveal zone, leading to build-up of fluid in the region manifested in the form of post-op CME.^4^,^5^ There is a well-established relationship between the intensity of post-op (cataract) inflammation and the development of severe CME reported in some studies.^6^ The above statement signifies that by lowering the post-operative inflammation the occurrence of CME can be reduced. Topical non-steroidal anti-inflammatory drugs (NSAIDs), alone or in combination with topical steroids, are widely used post-operatively in order to decrease the risk for CME development.^7^

Anterior chamber aqueous cells (AC-cells) measurement is used to grade the amount of the inflammatory reaction in AC, the level of which signifies the severity of blood aqueous barrier disruption.^8^,^9^ In this manner, AC-cells measurement may effectively evaluate the efficacy of topical Diclofenac and Nepafenac in altering the level of AC inflammation post-operatively and hence its impact on the CME development. To the best of our knowledge, limited studies have been conducted to date comparing two different NSAIDs by evaluating their responses on AC-cells.^1^,^3^ The rationale of the trial was to evaluate the therapeutic effectiveness of both NSAIDs in suppressing the AC-cells and its impact on preventing the development of post-op CME, which is an important cause of reduced vision in post cataract surgery patients.

## METHODS

**Fig.1:**
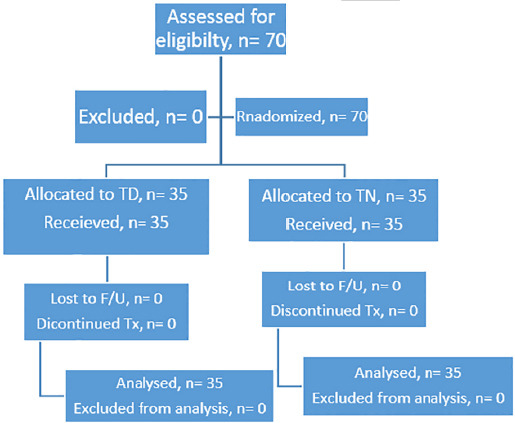
Flow chart showing the study methodology

A prospective, randomized control trial was conducted, at an Ophthalmology department of Qazi Hussain Ahmad Medical Complex, Nowshera from January till December 2021. We included a total of 70 patients in our trial, methodology of our study is shown in the Fig.1. The trial was conducted in accordance with the tenets of Declaration of Helsinki and guidelines of good clinical practice. The trial had been registered with Iranian registry of clinical trials (IRCT) with trial id IRCT20220607055097N2 at https://www.irct.ir. Written informed consent was acquired from all the participants of trial prior to the commencement of study.

### Ethical Approval:

The study was granted approval by the Institutional ethical review board (IERB) with No.0283 /R&D/IERB/NMC.

### Inclusion and Exclusion Criteria

Patients’ ≥ 50 yrs. Both males and females with visually significant senile-cataracts under-going phaco-emulsification with intra-ocular implants were selected.

We excluded patients with any past intra-ocular surgery, any history of intra-ocular inflammatory diseases, traumatic globe injuries, corneal disorders impairing the view, any glaucomatous diseases of eye, pseudo-exfoliation and maculopathies such as macular edema due to other etiologies or epi-retinal membranes or any age-related maculopathy. Those not willing to follow the study protocols and requirements of follow up were also excluded. Those who were pre-diabetics and confirmed diabetics, with systemic autoimmune/inflammatory disorders or any history of allergic reaction to the study drugs or other NSAIDs were also excluded. Oral steroids or NSAIDs intake was prohibited for the participants throughout the trial duration. Patients with eventful phaco surgery such as posterior-capsular tear, vitreous-prolapse or post-operative fibrinous reactions were not recruited for the study.

### Methods

All subjects underwent baseline evaluation a week before surgery. Pre-operative evaluation comprised of a detailed ophthalmic assessment including VA which was converted in log MAR, slit lamp examination, intra-ocular pressure (IOP) and biometry performed with Qantel Medical (France). AC-cells were counted under slit lamp with 1.6* magnification brought in with a slit beam of 1mm*1mm.^8^ Three consecutive readings were taken with the technique as mentioned above and average value was taken out as final measurement. The level of AC-cells was graded as shown in Table-I.^8^ Central macular thickness (CMT) was measured by spectral domain Optical coherence tomography (Topcon Inc. Japan) dense volume scan (15°×15°, approx. 5×5mm), 49 B-scans each spaced 110 μ apart, were obtained with the automatic real-time function operative.

**Table-I:** Standardization of Uveitis Nomenclature (SUN) Working Group Grading of Anterior chamber Cells (1mm by 1mm Slit Beam).

Grade	Cells in field
0	0
0.5	1-5
1	6-15
2	16-25
3	26-50
4	> 50

* Bowling B. Kanski, Clinical Ophthalmology. A systemic approach. In Uveitis. Elsevier publishing Co. 9^th^ ed; 2020; p 4278

At the day of the surgery, patients were randomly allocated to one of the two treatment arms by computer-generated randomization (topical diclofenac arm as TD and topical nepafenac group as TN arm). All the surgeries were done under peri-bulbar LA and performed by a single experienced senior surgeon, with the same phacoemulsification machine and surgical technique and all the patients underwent hydrophilic foldable Intaocular Lens (IOL) implantation.

At the end of the procedure an intra-cameral injection of 0.5 mg of moxifloxacin was given, and 0.5% moxifloxacin and 0.1% dexamethasone combination eye-drops (Deximox, Allergan Inc. USA) were commenced. At the time of discharge, all patients were put on Moxifloxacin 0.5% and dexamethasone 0.1% eye drops four times daily for a week followed by reduction of one drop each week as a tapering regimen.

Patients randomized to TD arm received diclofenac 0.1% eye drops solution (Diclomin^R^ 0.1%, Schazoo Inc. Pak) four times daily for four weeks, while TN arm received nepafenac 0.1% drops (Curanep^R^ 0.1%, Schazoo Inc. Pak) three times/day for four weeks. Post-operative full assessment was done by performing AC-cells measurement with slit lamp and CMT by spectral domain OCT on day 1^st^, 2^nd^, 4^th^ and 8^th^ week respectively. Statistical Analysis: Statistical analysis was done by using SPSS version 26.0 (IBM Corp. USA). Continuous/ quantitative variables were expressed as mean and standard deviation. As the data was not normally distributed so non-parametric tests were conducted to see for the significance of results.

For within the group analysis Wilcoxon signed rank test was performed and between the group analyses we used Mann Whitney test. For conducting correlational analysis between AC-cells and BC-VA non-parametric test Kendall’s tau-b was conducted. The statistical tests were set at less than 5% for significance of analysis.

## RESULTS

Seventy patients were recruited for the trial. All the participants successfully completed the entire follow-up. Two patients, one from TD and one from TN, developed CME with a markedly reduced VA. The mean age was 65.2 ± 6.4yrs. Forty were males (57.1%) and 30 were female patients (42.9%). (Table-II). Pre-operative and post-operative mean-VA in both arms is shown in Table-II. BC-VA improved in both groups by achieving a level of statistical significance (p <0.05), however, between the group analysis revealed statistically insignificant difference throughout the entire length of study (p>0.05). In Table-III Difference between pre-operative and post-operative IOP values at 2^nd^, 4^th^ and 8^th^ week was not statistically significant between the groups. On 1^st^ post-op day, AC-cells were significantly higher (20.15 ± 4.6 in TD and 19.44 ± 5.6 in TN) than before surgery (2.24 ± 1.5 in TD and 2.64 ± 1.6 in TN) in both arms (p<0.05). AC-cells reduced in the 4^th^ week (11.28 ± 5.04 in TD, 10.54 ± 4.05 in TN) and gradually reached pre-operative levels by 8^th^ week in both treatment arms. We observed a statistically significant difference (p<0.05) between the two arms in terms of AC-cells at two consecutive time points i.e., 2^nd^ and 4^th^ week post-operatively. In fact, AC-cells values in TN (10.54 ± 4.05 and 08.20 ± 4.44) were significantly lower than in TD (11.28 ± 5.04 and 9. 66 ± 5.50) respectively. (Table-IV). CMT didn’t change significantly throughout the length of study, with statistically insignificant difference between the two arms. (Table-V).

**Table-II:** Baseline characteristics of patients.

	TD (N=35)	TN (N=35)	p-value
Mean age in yrs. ± SD	64.6 ± 5.8	66.1 ± 5.4yrs	.242
Gender			
Male	20	18	
Female	15	17	
BC-VA	0.45 ± 0.4	0.46 ± 0.2	.246
CMT	280.2 ± 30.1	281.1 ± 26.4	.166
AC-cells	2.24 ± 1.5	2.64 ± 1.6	.128

BC-VA= Best corrected-visual acuity, CMT= central macular thickness, AC= Anterior chamber

**Table-III:** Mean BC-VA in Log MAR at follow-ups

	TD (N=35)	TN (N=35)	p-value
Baseline	0.45 ± 0.4	0.46 ± 0.2	.246
2^nd^ post-op week	0.03 ± 0.02	0.02± 0.05	.145
4^th^ post-op week	0.02 ± 0.04	0.02 ± 0.01	.562
8^th^ post-op week	0.01 ± 0.00	0.01 ± 0.02	.622

BC-VA= Best corrected-visual acuity, Log MAR= Log. Of minimum angle of resolution

**Table-IV:** Mean AC-cells in two treatment arms at follow-ups

	TD (N=35)	TN (N=35)	p-value
Baseline	2.24 ± 1.5	2.64 ± 1.6	.128
1^st^ post-op day	20.15 ± 4.6	19.44 ± 5.6	.122
2^nd^ post-op week	11.28 ± 5.04•	10.54 ± 4.05•	<.05
4^th^ post-op week	09. 66 ± 5.50•	08.20 ± 4.44•	<.05
8^th^ post-op week	2.20 ± 1.2	2.00 ± 1.5	.08

AC= Anterior Chamber, • p-value < 0.05

**Table-V:** Mean CMT measured by OCT in two treatment arms at follow-ups

	TD (N=35)	TN (N=35)	p-value
Baseline CMT in µm	280.2 ± 30.1	281.1 ± 26.4	.166
1^st^ post-op day CMT	280.9 ± 26.2	279.8 ± 28.2	.174
2^nd^ post-op week CMT	280.5 ± 30.1	280.0 ± 26.2	.082
4^th^ post-op week CMT	282.2 ± 24.5	281.8 ± 25.5	.106
8^th^ post-op week CMT	284.1 ± 26.6	283.8 ± 24.2	.092

CMT= central macular thickness, OCT= optical coherence tomography.

## DISCUSSION

In this trial, the comparative efficacy between TD 0.1% vs. TN 0.1% in suppressing the AC-cells after un-complicated Phacoemulsification revealed that both drugs were effective, but TN appeared to be more effective than TD at 2^nd^ and 4^th^ week post-op. Post-operative inflammation remained an important cause of delayed visual restoration among the patients who underwent cataract extraction.^7^ Numerous studies have been published regarding the efficacy of different agents utilized in suppression of post-operative inflammation.^1^,^3^,^4^,^6^ Steroids are commonly employed agents used post-operatively after intra-ocular procedures to control the inflammation by inhibiting the enzyme Phospholipase A2 that is elevated many folds post-op, in this way the level of arachidonic acid is reduced which acts as a potent inflammatory mediator.^10^ NSAIDs are also often prescribed post-operatively and numerous studies have shown that they are more effective than corticosteroids in preventing the disruption of blood retinal barrier post-op and hence it prevents the development of post-operative CME.^4^,^11^,^12^

Numerous trials have been done to discover the effective therapy that helps prevent the development of CME. Reports from various trials regarding the effectiveness of the different topical NSAIDs are still not yet clear and there are still gaps in the literature regarding the most effective topical NSAID which requires further comparative studies.^12^

Duan et al.^13^ did a systematic review by comparing different NSAIDs in terms of their efficacy and safety for the control of AC-cells post-operatively (cataract). They evaluated total of 12 studies, comparing the efficacy of various topical NSAIDs. They found out that, Diclofenac-0.1% was more effective in improving AC reaction post-op, whereas Nepafenac 0.1% was better at reducing the ocular pain. The pitfall of this review was that AC reaction was measured with flare-meter only in two of the studies taken for analysis. In one of them diclofenac-Na 0.1% was compared to ketorolac 0.5%, in the second one diclofenac-Na 0.1% to flurbiprofen 0.03%, and indomethacin 0.1%.^4^,^14^ Our findings were opposite to their observations of Diclofenac efficacy in suppressing post-operative inflammation. In our study AC-cells were significantly reduced at 2^nd^ and 4^th^ week post-op, in the TN treatment arm. Other researchers have studied the incidence of post-operative CME and/or changes in CMT during the follow-ups.^15^ In these cases, limitations can be due to the fact that there is still a lack of consensus in defining significant macular post-operative changes. Furthermore, macular thickness fluctuation is an indirect index of intra-ocular inflammation which can be governed by many factors such as ocular diseases/therapies, pre-operative and post-operative medications and systemic conditions.^15^,^16^ Another study reported the efficacy of topical nepafenac as post-operative therapy in suppressing the AC inflammation as compared to other classes of NSAIDs.^3^ We noted that during the 1^st^ few weeks, post-op TN appeared more effective than TD, which is widely used and safe NSAID. In support of other studies done we observed AC-cells level almost similar eight weeks post-operatively to the baseline in both arms, with statistically insignificant difference between groups. In our study, different levels of AC-cells at the 4^th^ week post-op didn’t have any clinical consequences. We didn’t notice any significant difference in VA and CMT during the entire length of study between the two groups. These findings are in agreement with other published studies, showing that reduced post-operative inflammation didn’t correlate with better VA.^3^,^17^ However, such findings should not underestimate the significance of controlling the post-operative (cataract) inflammation in the 1^st^ few weeks because this is the most likely factor for the development of CME and visual deterioration.^1^-^7^,^9^,^11^-^15^ In corroboration with the above statement, two patients of our population developed CME with increased AC-cells during the entire post-operative length of study. Our findings, are supported by the findings from other studies for stringent control of post-operative inflammation to reduce the incidence of CME.^9^

In contrast to our study observation, Ylinen et al.^17^ reported no difference between nepafenac 0.1% and diclofenac 0.1% in controlling post-operative inflammation (post-op cataract surgery). The disagreement may be attributed to many factors like sample size, patient demographics, methodology and frequency of doses (eye drops) used in the subjects. Furthermore, he recruited patients with systemic/ocular diseases such as pseudo-exfoliation syndrome (PXF), diabetes or sporadic use of systemic anti-inflammatory drugs. However, we excluded such patients from our study. Interestingly one study reported that ocular as well as systemic inflammatory conditions can influence post-operative AC-cells value, as in cases of PXF.^18^

A local study conducted by Sarfraz et al.^19^ on the efficacy of topical Nepafenac 0.1% in prevention of pseudophakic cystoid macular edema in diabetic patients, concluded that the incidence of macular edema was 3.3% as compare to those patients not receiving it i.e., 23.3%, this difference was statistically significant, the author described this effect due the anti-inflammatory effect of nepafenac both in the anterior as well as posterior chamber of the eye. Another local study by Ullah et al.^20^ compared two groups one was given topical nepafenac 0.1% along with standard post-op medications (steroids and antibiotics) while other group was only given standard post op regimen only in post phacoemulsification patients, the results showed that macular thickness was statistically less (p < 0.01) in nepafenac group versus standard regimen only, the above two local studies support our study finding of reduced macular edema post operatively in topical nepafenac due to its enhanced anti-inflammatory efficacy. Similar to the above two studies yet another study done locally by Amjad et al.^21^ supporting the findings derived from the above studies by showing the significance of topical nepafenac in reducing the macular thickness (edema) in post cataract surgery diabetic patients but without retinopathy versus those patients not being given nepafenac, the comparison was done at 1^st^ and 4^th^ week post operatively with statistically significant difference. It may be due to its better penetration, bio availability and pharmacokinetics as compared to diclofenac making it more effective in suppressing the inflammation.

### Limitations

It includes small sample size, lack of control group, limited follow up period, lack of blinding in the study. Further prospective, multi-center randomized control trials are needed to further explore any gaps in knowledge in this area to better understand the usefulness of these agents in post-operative regimen.

## CONCLUSIONS

Topical Nepafenac 0.1% was more effective in suppressing the anterior chamber inflammation as compared to diclofenac during the 1^st^ few weeks post-operatively. It may be due to its better penetration, bio availability and pharmacokinetics as compared to diclofenac making it more effective in suppressing the inflammation

### Author’s Contribution:

**AA:** Study design, concept, data acquisition & interpretation.

**SUH:** Data acquisition & interpretation, critical revision and editing.

**JH:** Statistical analysis, literature search, final draft preparation.

**JR:** Critical revision, editing of manuscript, final draft.

All of the above authors are responsible and accountable for the accuracy or integrity of the work.
